# Comparative effects of free doxorubicin, liposome encapsulated doxorubicin and liposome co-encapsulated alendronate and doxorubicin (PLAD) on the tumor immunologic milieu in a mouse fibrosarcoma model

**DOI:** 10.7150/ntno.75045

**Published:** 2022-09-01

**Authors:** Md. Rakibul Islam, Jalpa Patel, Patricia Ines Back, Hilary Shmeeda, Konstantin Adamsky, Hui Yang, Carlos Alvarez, Alberto A. Gabizon, Ninh M. La-Beck

**Affiliations:** 1 Department of Immunotherapeutics and Biotechnology, Texas Tech University Health Sciences Center School of Pharmacy, Abilene, TX, USA.; 2 Oncology Institute, Shaare Zedek Medical Center and Hebrew University-Faculty of Medicine, Jerusalem, Israel.; 3 Levco Pharmaceuticals Ltd., Jerusalem, Israel.; 4 Department of Pharmacy Practice, Texas Tech University Health Sciences Center School of Pharmacy, Abilene, TX, USA.

**Keywords:** chemotherapy, immunotherapy, nanomedicine, bisphosphonate, tumor-associated macrophages, alendronate, doxorubicin, fibrosarcoma

## Abstract

**Background:** We have previously shown that alendronate, an amino-bisphosphonate, when reformulated in liposomes, can significantly enhance the efficacy of cytotoxic chemotherapies and help remodel the immunosuppressive tumor microenvironment towards an immune-permissive milieu resulting in increased anticancer efficacy. In addition, we have previously shown that the strong metal-chelating properties of alendronate can be exploited for nuclear imaging of liposomal biodistribution. To further improve anticancer efficacy, a pegylated liposome formulation co-encapsulating alendronate and doxorubicin (PLAD) has been developed. In this study, we examined the effects of PLAD on the tumor immunologic milieu in a mouse fibrosarcoma model in which the tumor microenvironment is heavily infiltrated with tumor-associated macrophages (TAM) that are associated with poor prognosis and treatment resistance.

**Methods:** Doxorubicin biodistribution, characterization of the tumor immunologic milieu, cellular doxorubicin uptake, and tumor growth studies were performed in Balb/c mice bearing subcutaneously implanted WEHI-164 fibrosarcoma cells treated intravenously with PLAD, pegylated liposomal doxorubicin (PLD), free doxorubicin, or vehicle.

**Results:** PLAD delivery resulted in a high level of tumor doxorubicin that was 20 to 30-fold greater than in free doxorubicin treated mice, and non-significantly higher than in PLD treated mice. PLAD also resulted in increased uptake in spleen and slightly lower plasma levels as compared to PLD. Importantly, our results showed that PLAD, and to a lesser extent PLD, shifted cellular drug uptake to TAM and to monocytic myeloid-derived suppressor cells (MDSC), while there was no drug uptake in neutrophilic MDSC or lymphoid cells. Free doxorubicin cellular drug uptake was below detectable levels. PLAD, and to a lesser extent PLD, also induced significant changes in number and functionality of tumor-infiltrating TAM, MDSC, Treg, NKT, and NK cells that are consistent with enhanced antitumor immune responses in the tumor microenvironment. In contrast, free doxorubicin induced moderate changes in the tumor microenvironment that could promote (decreased Treg) or be detrimental to antitumor immune responses (decreased M1 TAM and NK cells). These immune modulatory effects are reflected in the therapeutic study which showed that PLAD and PLD inhibited tumor growth and significantly prolonged survival, while free doxorubicin showed little or no anticancer activity.

**Conclusion:** We show that liposomal delivery of doxorubicin not only alters pharmacokinetics, but also dramatically changes the immune modulatory activity of the drug cargo. In addition, our data support that the PLAD nanotheranostic platform further enhances some immune changes that may act in synergy with its cytotoxic chemotherapy effects.

## Introduction

Fibrosarcoma is one of the main subtypes of a group of malignant tumors known as soft tissue sarcomas that occurs in infants and adults alike 1, 2. This tumor usually develops within the deep soft tissues adjacent to the bone and around the skeletal muscles. Primary treatment for the early stages of the disease is surgical intervention in combination with radiotherapy. However, soft tissue sarcomas, and particularly fibrosarcoma, often recur locally and metastasize primarily, but not only, to the lungs. Systemic drug therapy is required for surgically unresectable and metastatic fibrosarcoma 3-5. Doxorubicin, a widely used cytotoxic drug that works primarily by intercalating DNA base pairs and inhibiting topoisomerase II 6, is the standard of care and one of the most effective chemotherapies for these patients. However, the response rate is low and, in most cases, short-lived 7, 8, while toxicity is severe, particularly irreversible cumulative cardiac toxicity 9, 10. One of the effective ways to diminish the adverse events associated with doxorubicin is its encapsulation in pegylated liposomes. Pegylated liposomal doxorubicin (PLD) alters the pharmacokinetic profile of doxorubicin which results in a longer half-life and modified tissue distribution of the drug. Consequently, PLD has reduced cardiotoxicity and improved tolerability over free doxorubicin (F-Dox) 8, 11. This is apparently due to selective accumulation of the liposomal carrier in tumor tissues through the enhanced permeability and retention (EPR) effect 12, 13.

The tumor microenvironment of soft tissue sarcomas is highly infiltrated with tumor associated macrophages (TAM) and other myeloid-derived immune cells along with lymphoid cells 14-16. The presence of TAM in large numbers within the tumor microenvironment has been found to be associated with poor patient prognosis and treatment resistance across different cancer types 17. In patients with soft tissue sarcoma, TAM infiltration has been linked to sustained immunosuppression and angiogenesis that promote tumor growth and metastasis 18, 19. Moreover, inflammatory cytokines such as IL-6 and TNF-α that are secreted by TAM can result in resistance to chemotherapies, and the selective reduction of TAM in the tumor microenvironment increases effectiveness of chemotherapeutic agents 17, 19-21. To counter the complex effects of TAM on the tumor microenvironment, multiple therapeutic strategies are being explored in various tumor models. Anti-angiogenic therapies such as bispecific antibody against angiopoietin-2 and vascular endothelial growth factor can remodel TAM to an anti-tumoral phenotype 22, 23. Some studies suggest that a combination of immune checkpoint inhibitors with chemotherapies might be efficacious to curb TAM-driven immunosuppression and obtain responses in soft tissue sarcomas 24, 25. Certain chemotherapies alone, such as trabectedin and paclitaxel, have also been reported to improve immune competence in the tumor microenvironment and polarize pro-tumoral TAM to anti-tumoral phenotypes 26, 27. Repurposing antiresorptive bisphosphonates, which can exert cytotoxic effects on myeloid cells such as TAM, to treat several cancer types have been explored 23. When encapsulated in liposomal carriers, the first-generation bisphosphonates (e.g., clodronate) deplete systemic macrophages as well as TAM to reduce angiogenesis 28. However, the second-generation bisphosphonates (e.g., alendronate), known as amino-bisphosphonates, are more useful and specific tools that reduce immunosuppression in the tumor microenvironment by polarizing TAM towards an anti-tumoral phenotype 29 and activating a specific subset of T cells with natural antitumor properties 30. When alendronate is given as free drug, it is rapidly eliminated renally with distribution mainly to osseus tissue where it interrupts bone resorption by inhibiting the mevalonate pathway in osteoclasts 31, 32. When alendronate is stably encapsulated in pegylated liposomes (PLA), it drastically alters pharmacokinetic and pharmacologic profiles compared to free alendronate, including increased half-life and drug distribution to tumor tissue 29. PLA has been found to activate various effector T cell populations and inhibit protumoral macrophages in the tumor microenvironment 32. Furthermore, PLA has been shown to be a useful imaging agent of liposome biodistribution due to the strong radiometal-chelating properties of alendronate and its stable encapsulation in liposomes 33.

**Figure 1:**
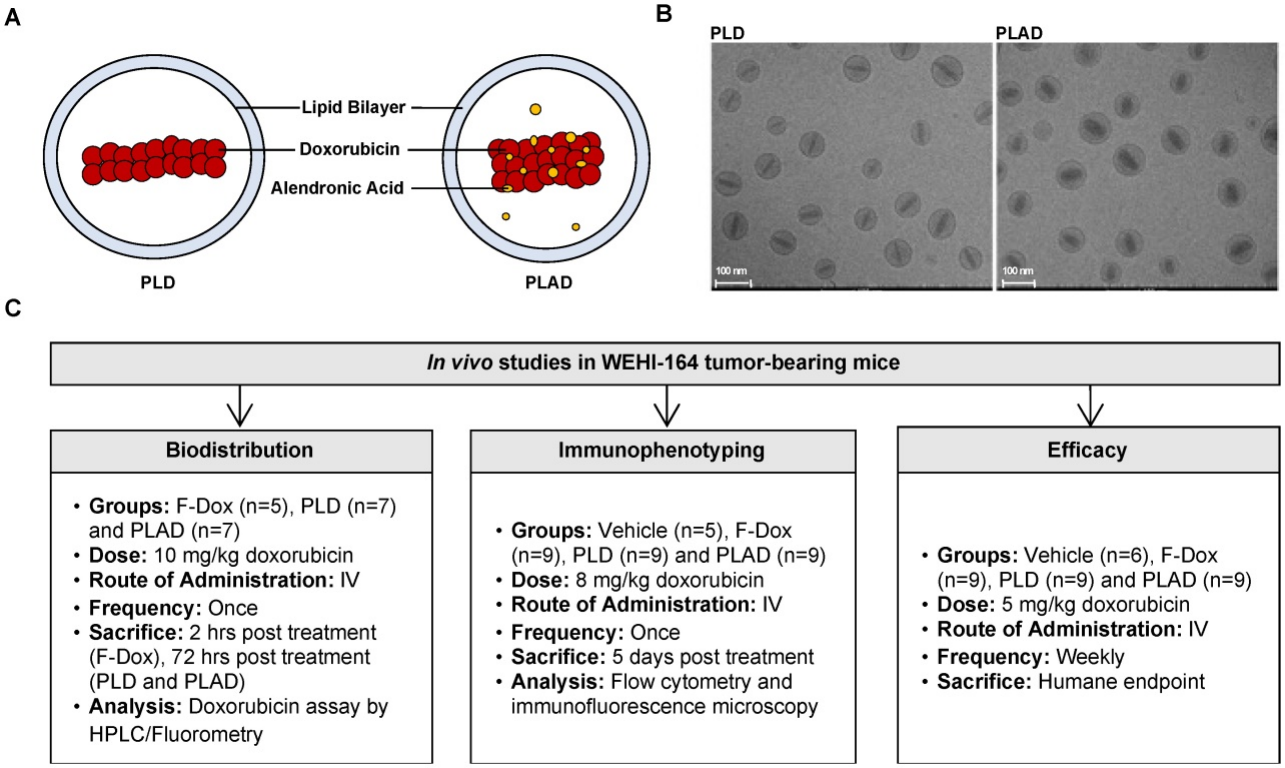
** Experiment Schema. (A)** Pictograms and **(B)** cryoTEM images of PLD and PLAD liposomes. **(C)** Overview of the three *in vivo* studies.

We therefore hypothesized that doxorubicin could be co-encapsulated with alendronate, resulting in a formulation coined PLAD, which would provide a nanotheranostic strategy targeting both tumor immune dysfunction and tumor proliferation pathways to inhibit the growth of fibrosarcoma and other tumors 34. In this study, we characterized the changes in the tumor immunologic milieu in response to F-Dox, PLD, and PLAD therapy, as well as their *in vivo* tissue and cellular distribution and antitumor efficacy in a murine fibrosarcoma model (Figure 1).

## Methods

### Formulation of PLAD

The liposome components and their sources were: hydrogenated soybean phosphatidyl-choline (HSPC) (Lipoid, Germany), methoxy-polyethylene glycol-distearoyl-phosphatidylethanolamine (mPEG_2000_-DSPE) (Bio-lab, Jerusalem, Israel), cholesterol (Chol), ammonium hydroxide (Sigma, St. Louis, MO), alendronic acid (Tokyo Chemical Industry Co Ltd., Japan), and doxorubicin (Teva, Israel). Pegylated liposomal doxorubicin (PLD) from Janssen Pharmaceuticals, NJ (commercial name Doxil^TM^ or Caelyx^TM^) and free doxorubicin HCl (F-Dox) (Teva, Israel) were used as comparators.

PLAD liposomes were provided by Levco Pharmaceuticals (Jerusalem, Israel) and were prepared at Nextar Chempharma (Ness Ziona, Israel) by the standard method of ethanol injection into an aqueous buffer containing alendronate for passive encapsulation, followed by extrusion, buffer exchange, and remote loading for encapsulation of doxorubicin based on a method previously described 34 with modifications and adjustments to larger batch size. Briefly, the lipid components: HSPC, mPEG_2000_-DSPE, and Chol at molar ratios of 55%, 5% and 40%, respectively, were weighed and dissolved in ethanol. The aqueous buffer was prepared by mixing a solution of alendronic acid with a concentrated solution of ammonium hydroxide (25%) in the amounts required to obtain a 250 mM salt of ammonium alendronate. The final pH was in the range of 6.5-7.0. After mixing and shaking for 1 hour at 60 °C, the resulting multilamellar vesicles were downsized by repeated extrusion in a high-pressure extruder device (Lipex Biomembranes, Vancouver, BC), with temperature controlled at 60 °C, through double stacked polycarbonate membrane filters of 0.08 µm pore size. Nonencapsulated alendronate was removed by tangential flow filtration (TFF) against a buffer of 5% dextrose with 17 mM sodium HEPES, at pH 7.0. The liposomes were then incubated for 30 min at 60 °C with a solution of 10 mg/mL doxorubicin HCl in 5% dextrose, at alendronate/doxorubicin molar ratios ranging between 1 and 1.5. The doxorubicin-loaded liposomes were again processed by TFF to remove any nonencapsulated doxorubicin, and sterilized by filtration through 0.22 µm-pore cellulose membranes. Before sterile filtration, the final concentration of doxorubicin in the formulation was adjusted to 1.0 mg/mL by further dilution with the dialysis buffer (5% dextrose/17 mM HEPES). The concentrations of the liposome components and characteristics of PLAD are presented in supplemental information (Supplemental Table S1).

All experiments in this study were conducted based on the doxorubicin content of PLAD, which was measured by an HPLC assay previously described 34. PLAD average vesicle size, as measured by dynamic light scattering, was 90-100 nm with narrow polydispersity (PDI<0.15).

### Animal model

Male Balb/c mice (8-10 weeks old from Jackson Labs) were implanted sc with 3 × 10^6^ WEHI-164 fibrosarcoma cells (passage 6, >95% viability). When average tumor size reached ~300 mm^3^, animals were randomized 1:1:1 to receive a single iv injection of PLAD, PLD, or F-Dox, at doses equivalent to 8 mg/kg of doxorubicin (n=9 each); the vehicle control group (n=5) was injected with 5% dextrose at equivalent volume. Animals were euthanized and tumor tissue harvested for immunophenotyping by multi-parameter flow cytometry at 5 days post treatment administration. If there was sufficient tissue, tumors were also processed for evaluation of doxorubicin by fluorescence microscopy.

To assess antitumor efficacy of PLAD, male Balb/c mice were implanted sc with WEHI-164 fibrosarcoma cells as above. When average tumor size reached 150-200 mm^3^, animals were randomized 1:1:1 (n=9 each) to receive weekly iv injection of PLAD, PLD, or F-Dox, at doses equivalent to 5 mg/kg of doxorubicin, containing approximately 2.5 mg/kg alendronate for PLAD; an additional control group was 5% dextrose vehicle at equivalent volume (n=6). Tumor volume was monitored by digital caliper at least twice weekly, and animals were euthanized when humane endpoints were reached. All animals in this therapeutic study and in the above immunophenotyping study were used in accordance with a protocol approved by the Institutional Animal Care and Use Committees of the Texas Tech University Health Sciences Center.

To assess *in vivo* doxorubicin biodistribution, the same mouse model was used as above. In this experiment, BALB/c mice bearing WEHI-164 tumor implants were injected iv with F-Dox (n=5), PLD (n=7), and PLAD (n=7) at a dose of 10 mg/kg based on the doxorubicin concentration. Since F-doxorubicin disappears very fast from circulation and reaches maximal distribution to tissues within 1-2 hours, mice injected with F-Dox were bled and euthanized for tissue collection 2 hours after injection, while PLD and PLAD-injected mice were bled and euthanized 72 hours after injection. Prior to bleeding, mice were anesthetized by isoflurane inhalation. Blood was collected into heparinized tubes and centrifuged to recover plasma which was frozen and stored at -70 °C. Liver, spleen, kidneys, and tumor were dissected out, weighed, and stored at -70 °C. This study was approved by the Animal Ethics Committee of the Hebrew University of Jerusalem.

### Flow cytometry

Tumors were excised, minced, and incubated for enzymatic digestion (digestion buffer contains Liberase^TM^ Research Grade, cat#5401119001 and DNase 1 cat#10104159001, both from Sigma Aldrich) for 20 min, then neutralized with media containing FBS and passed through 40 µm strainers to obtain single cell suspensions. Red blood cells were lysed with ACK lysis solution. Single cell suspensions were counted and viability assessed using trypan blue exclusion assay (Vi-Cell XR, Beckman Coulter Inc. California, USA).

Three million cells from each sample were stained with antibodies against cellular surface markers to identify cell subpopulations and characterize their activation/polarization states. After surface staining, cells in the T-regulatory panel were fixed, permeabilized, and stained for FoxP3. Details of the antibodies and other reagents are in Supplemental Tables S2-S4. Stained samples were analyzed on a BD LSR Fortessa flow cytometer (BD Biosciences, San Jose, CA) and one million events were acquired for each sample. Compensation and analyses were performed using FlowJo software (Tree Star Inc., Ashland, Oregon, USA). Briefly, single cells were gated using the forward scatter and side scatter parameters, followed by dead cell exclusion via a fixable viability dye (Biolegend cat#423104). Antibody staining panels were designed to enable identification of: TAM (CD11b^+^F4/80^+^), monocytic myeloid-derived suppressor cells (MDSC) (CD11b^+^Gr1^dim^F4/80^-^), neutrophilic MDSC (CD11b^+^Gr1^hi^F4/80^-^), conventional dendritic cells (DC2) (CD8a^-^CD11b^+^), antigen cross presenting dendritic cells (DC1) (CD8a^+^CD11b^-^), natural killer cells (CD3^-^NKp46^+^CD49^+^), natural killer T cells (CD3^+^NKp46^+^CD49^+^), cytotoxic T cells (CD3^+^CD8^+^CD4^-^), helper T cells (CD3^+^CD8^-^CD4^+^), and T regulatory cells (CD4^+^FoxP3^+^). Doxorubicin fluorescence is in the PE-TR channel with some spillover into the PE channel, therefore no antibodies conjugated to these fluorophores were used in the myeloid panel so that intracellular doxorubicin uptake could be assessed in TAM and MDSC, and in non-myeloid leukocytes (i.e., lymphocytes). Preliminary studies were performed to optimize the FACS procedures and verify that there was no interference between cellular doxorubicin and fluorescent markers in the adjacent detection channels.

### Fluorescence microscopy

A portion of the above tumors were also collected and mounted in OCT and stored at -80 °C protected from light until sectioning. Tumor sections 5 µm thick were immediately counterstained with DAPI and images acquired at 20X (NIKON A1 microscope) for doxorubicin fluorescence. Doxorubicin was detected in both the FITC and TRITC channels; FITC channel was used for doxorubicin quantification (NIS Element software) since the signal to noise ratio was better than in the TRITC channel. Doxorubicin accumulation was determined as the sum of the positive area for each tumor section image.

### Doxorubicin bioanalysis

Extraction and quantitation of doxorubicin from plasma and tissues were done following previously published methods 35. Plasma levels of doxorubicin were measured fluorometrically (Ex=470, Em=590 nm) after extraction of doxorubicin from plasma with acidified (HCl 0.075 N) isopropanol. For tissue levels of doxorubicin, a two-phase drug extraction procedure from homogenized tissues with a daunorubicin internal standard followed by reverse phase HPLC with fluorometric detection was done as previously described 36.

### Statistical analyses

Unpaired t-test was used to compare two groups, one-way ANOVA was used to compare more than two groups, and two-way ANOVA was used to compare tumor volume and cellular doxorubicin uptake among different TAM populations (GraphPad Prism version 8.0 or higher). For tumor volume, the two factors were time and treatment, and for doxorubicin uptake, the two factors were TAM polarization state and treatment. If ANOVA was significant, then post-hoc tests were performed with correction for multiple comparisons. Survival was analyzed using the Kaplan-Meier method and compared statistically using the log-rank test in SAS 9.4. The survival endpoint was defined as 5-fold tumor growth from baseline tumor volume at time of treatment initiation. For the survival analysis, mice were censored if they 1) experienced a 5-fold increase in tumor burden, 2) were moribund and sacrificed due to reasons specified in the protocol, or 3) the end of the study period at 50 days. The primary objective was to demonstrate superior efficacy of PLAD versus F-Dox, the clinically approved standard of care of soft tissue sarcomas; secondary objectives included a comparison of PLD versus F-Dox, and PLAD versus PLD. For all analyses, alpha was set at 0.05 and p < alpha was considered statistically significant.

## Results

### *In vivo* tissue distribution

**Figure 2:**
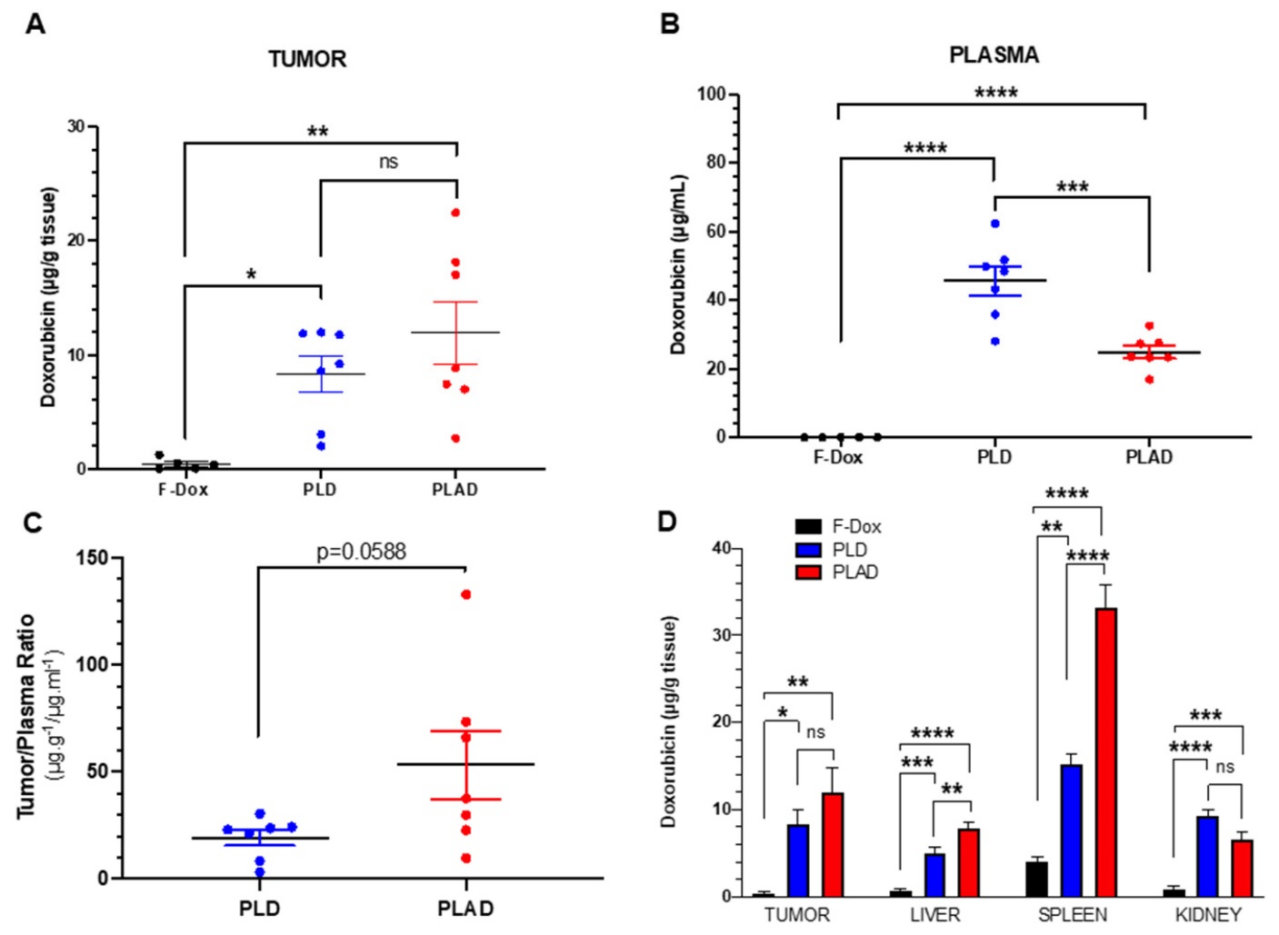
** PLAD, PLD and F-Dox biodistribution in WEHI-164 tumor bearing mice at 72 h (PLAD, PLD) and 2 h (F-Dox) post-dose.** Data are mean with SEM; n=7 for PLD and PLAD, n=5 for F-Dox. ANOVA with Tukey's test **(A, B, D)** or unpaired t-test **(C)**; *p<0.05, **p<0.01, ***p<0.001, ****p<0.0001, ns: not significant. F-Dox: free doxorubicin, PLD: pegylated liposomal doxorubicin, PLAD: pegylated liposomal alendronate-doxorubicin.

PLD- and PLAD-injected mice had significantly higher doxorubicin concentrations in tumor, spleen, liver, kidney, and plasma compared to F-Dox at 2 hours post dose (Figure 2). Both liposome formulations increased tumor doxorubicin concentration over F-Dox (Figure 2A). There was a non-significant increase of the average tumor doxorubicin levels when PLAD was compared to PLD despite the almost 2-fold higher plasma levels in PLD-dosed mice (Figure 2A-C). Tumor doxorubicin distribution for PLAD appeared to be polarized into two groups with one group (n=4) very similar to PLD and the other group (n=3) showing higher tumor doxorubicin levels than PLD (Figure 2A). The values of % injected dose/gram in 3/7 of the PLAD injected mice reached ~10%. Since our results were not corrected for blood tissue content, it is likely that the real difference between PLD and PLAD is somewhat greater than what we measured. The doxorubicin concentrations in spleens from PLAD-injected mice were very high, as observed before 34, even higher than spleens of PLD-injected mice (Figure 2D).

### Modulation of tumor immunologic milieu

**Figure 3:**
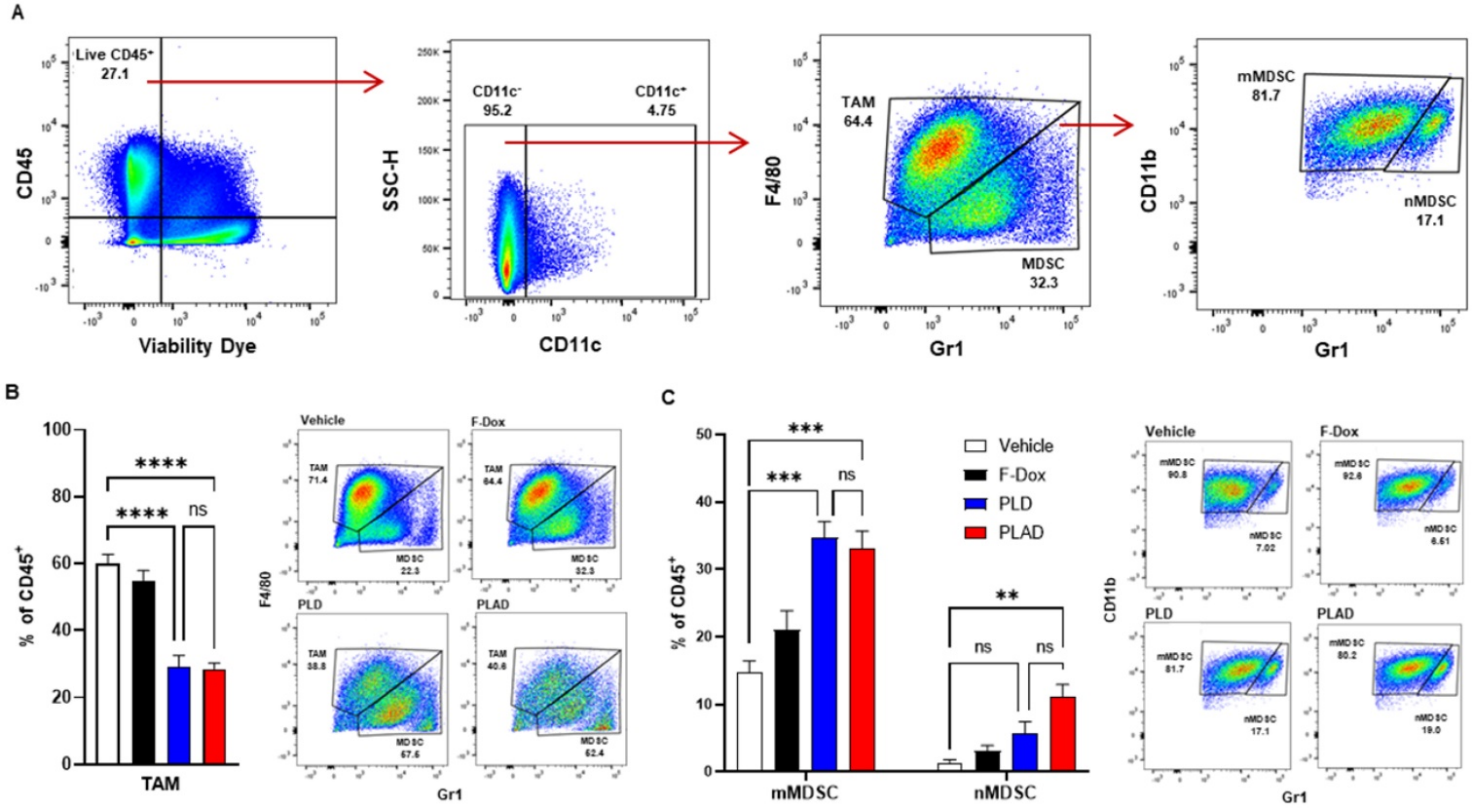
** PLAD and PLD reduced TAM and increased monocytic MDSC in the tumor microenvironment. (A)** Gating strategy for TAM, mMDSC, and nMDSC. **(B)** PLAD and PLD significantly decreased the number of TAM and **(C)** increased mMDSCs in tumors. PLAD also increased nMDSC but the other treatments did not. Representative FACS plots are shown. Data are mean with SEM, n=9 for PLAD, PLD and F-Dox, n=5 for vehicle; ANOVA with Tukey's test; **p<0.01, ***p<0.001, and ****p<0.0001. F-Dox: free doxorubicin, PLD: pegylated liposomal doxorubicin, PLAD: pegylated liposomal alendronate doxorubicin, TAM: tumor associated macrophages, mMDSC: monocytic myeloid derived suppressor cells, nMDSC: neutrophilic myeloid derived suppressor cells.

**Figure 4:**
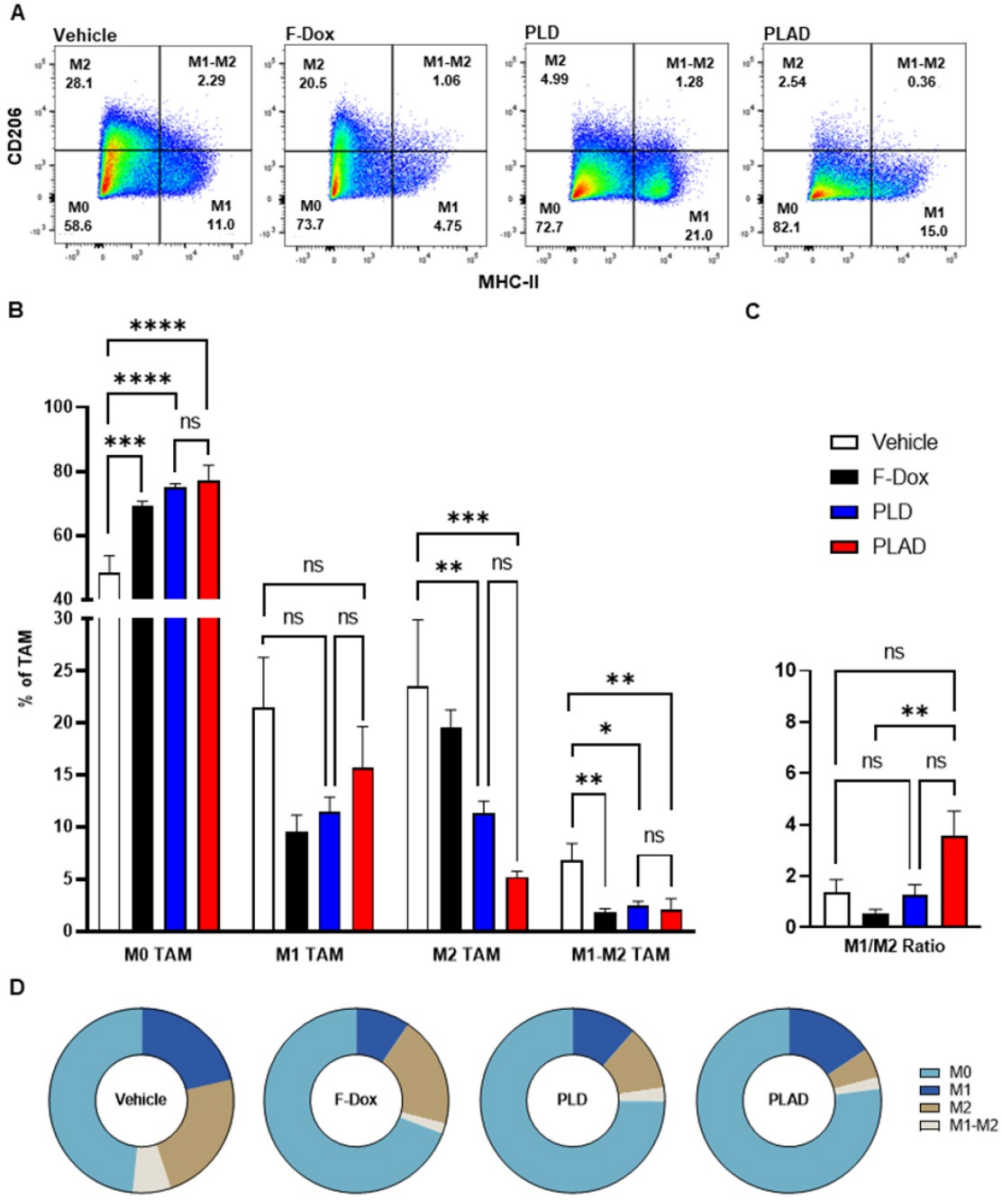
** Effects of doxorubicin on TAM polarization depends on liposomal drug delivery and alendronate co-encapsulation. (A)** TAM polarization was determined in the TAM population from Figure 3A. **(B)** All treatments significantly increased non-polarized M0 TAM. Free doxorubicin treatment decreased M1 TAM, while PLD and PLAD had no effects on this population. M2 polarized TAM were decreased in PLD and PLAD groups with the greatest effect in PLAD, although the difference between PLD and PLAD was not significant. All treatments also decreased M1-M2 TAM, a population that expresses both M1 and M2 markers. **(C-D)** PLAD significantly increased M1/M2 ratio compared to F-Dox. Representative FACS plots are shown. Data are mean with SEM, n=9 for PLAD, PLD and F-Dox, n=5 for vehicle; ANOVA with Tukey's test; *p<0.05, **p<0.01, ***p<0.001, and ****p<0.0001. F-Dox: free doxorubicin, PLD: pegylated liposomal doxorubicin, PLAD: pegylated liposomal alendronate doxorubicin, TAM: tumor associated macrophages.

The median and mean tumor volumes were not significantly different between groups at time of dosing and at time of tissue collection, 5 days post dosing (Supplemental Figure S1). There was no statistically significant difference in overall numbers of tumor-infiltrating leukocytes between the treatment groups (Supplemental Figure S2). However, inspection of specific immune cell populations revealed that PLD and PLAD significantly decreased the number of TAM, while F-Dox did not (Figure 3A-B). PLD and PLAD both increased monocytic MDSC, while only PLAD increased neutrophil MDSC (Figure 3C). This could be due to inhibition of MDSC differentiation into TAM or tumor-associated neutrophils (TAN), or it could indicate a highly inflammatory tumor microenvironment. In control mice, more than half of the TAM were activated and the proportion of M1-polarized TAM was roughly equal to M2-polarized TAM (Figure 4A-B). F-Dox, PLD, and PLAD all inhibited TAM polarization as indicated by an increase in the M0 population. M1 and M2 TAM were both decreased in the PLD group, but the M1/M2 ratio remained roughly equal to vehicle control (Figure 4C-D). In contrast, PLAD decreased M2 TAM but not M1 TAM, resulting in an increase in the M1/M2 ratio (Figure 4C-D), suggesting more antitumoral polarization of the TAM population. F-Dox treatment decreased M1 TAM but not M2 TAM, resulting in a decrease in M1/M2 ratio (Figure 4C-D) which could indicate more protumoral polarization of the TAM population.

**Figure 5:**
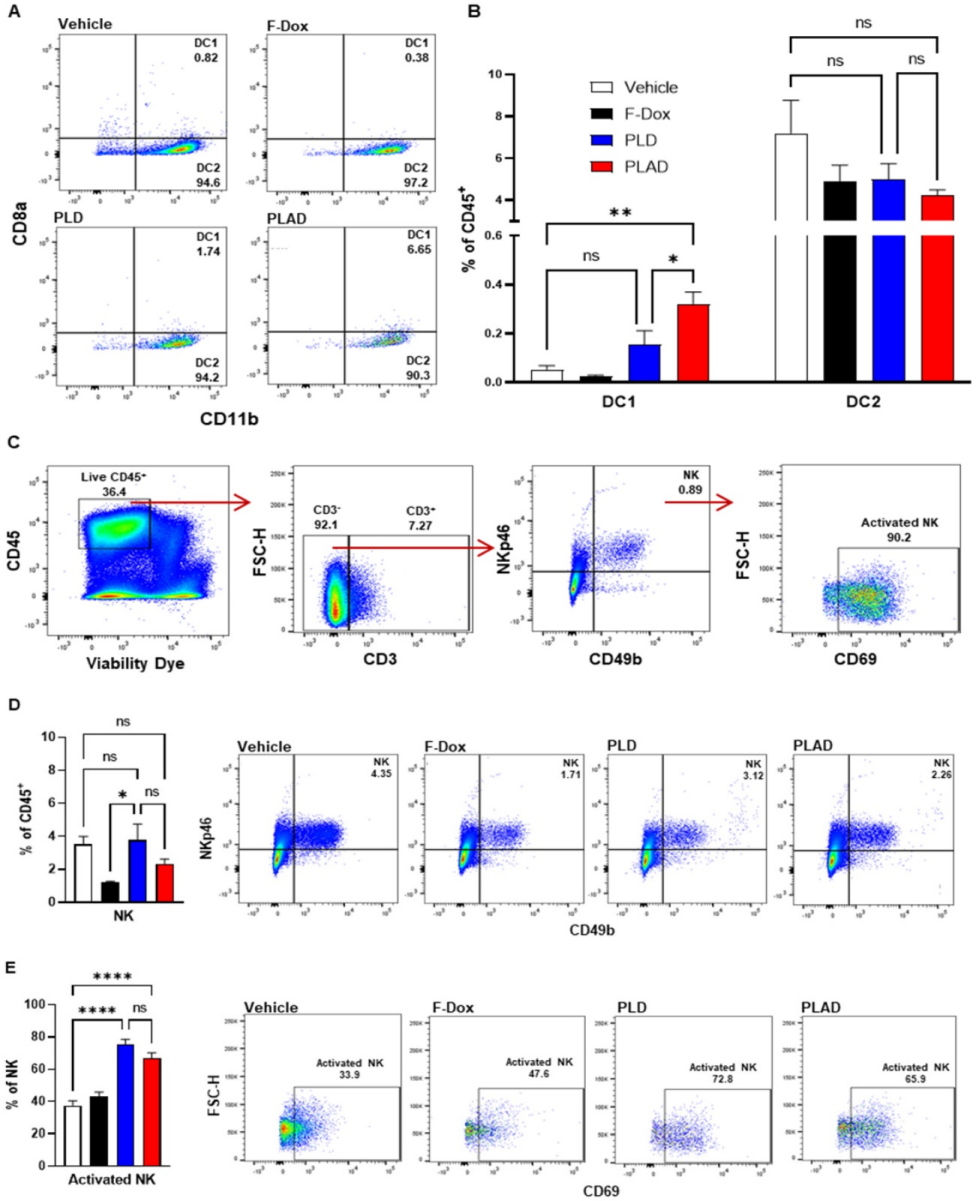
** PLAD increased antigen cross presenting dendritic cell infiltration and NK cell activation in tumors. (A-B)** Antigen cross presenting dendritic cells (DC1) and conventional dendritic cells (DC2) were gated from the CD11c^+^ population in Figure 3A. PLAD increased tumor infiltration of DC1, while DC2 infiltration was not affected by any treatment. **(C)** Gating strategy for NK cells. **(D)** Although NK cell infiltration in the tumor microenvironment was not affected, **(E)** PLD and PLAD increased the proportion of activated NK cells. Representative FACS plots are shown. Data are mean with SEM, n=9 for PLAD, PLD and F-Dox, n=5 for vehicle; ANOVA with Tukey's test; *p<0.05, **p<0.01, and ****p<0.0001. F-Dox: free doxorubicin, PLD: pegylated liposomal doxorubicin, PLAD: pegylated liposomal alendronate doxorubicin, DC: dendritic cells, NK: natural killer cells.

**Figure 6:**
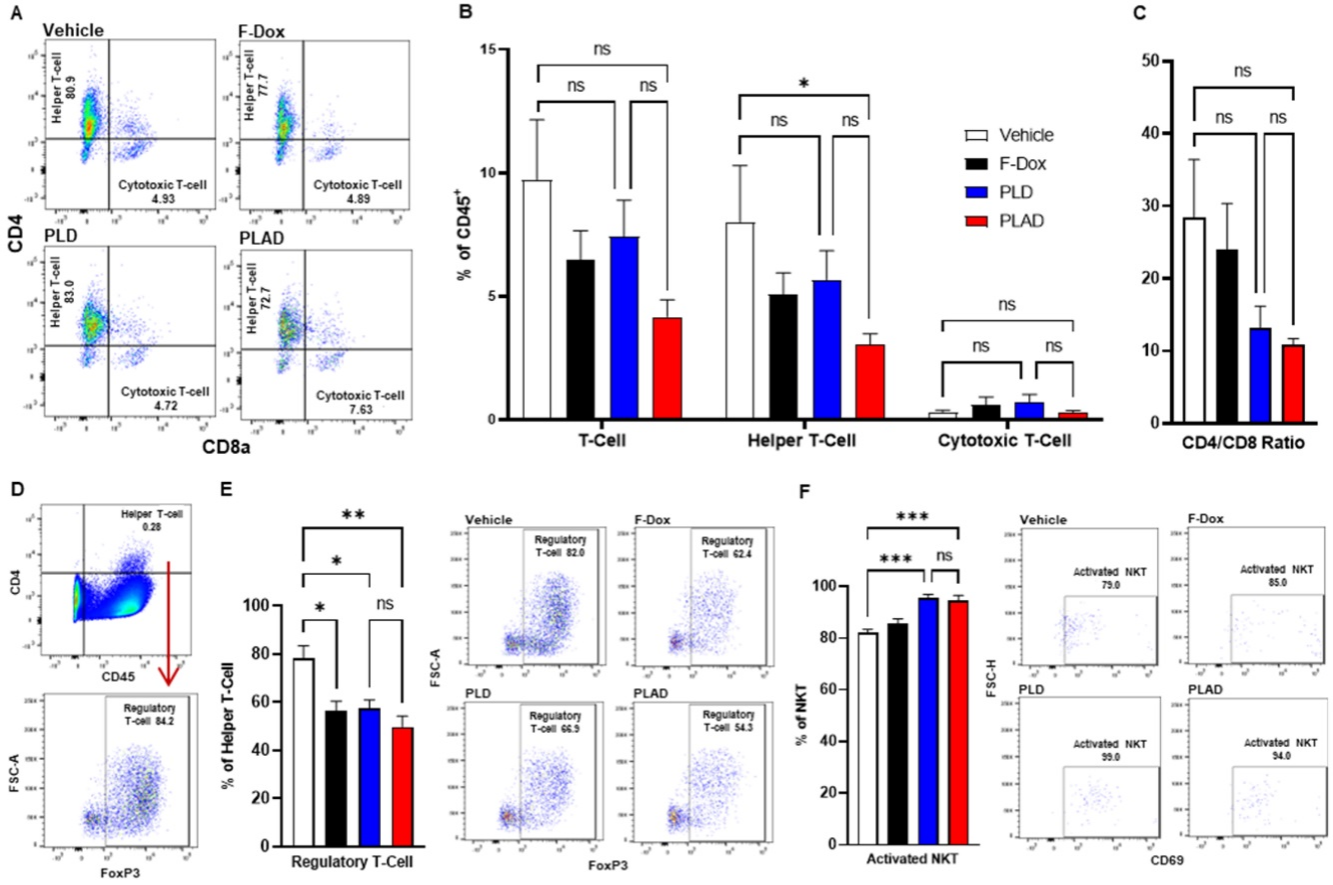
** PLAD decreased T regulatory cells and increased activated NKT cells in tumors. (A-B)** Helper and cytotoxic T cells were identified from the CD3^+^ population in Figure 5C. There were no significant differences in the total tumor infiltrating T cell population. However, further inspection showed that there was a significant decrease in helper T cells in PLAD treated animals. There was no significant impact on cytotoxic T cells for any treatment. **(C)** PLAD showed a decreased CD4/CD8 ratio, but it was not statistically significant. **(D)** T regulatory cells were gated from live cells (Supplemental Figure S4). **(E)** PLAD, PLD, and F-Dox significantly decreased the infiltration of regulatory T cells in tumors. **(F)** PLAD and PLD also increased the activation of NKT cells in tumors. Representative FACS plots are shown. Data are mean with SEM, n=9 for PLAD, PLD, and F-Dox, n=5 for vehicle; ANOVA with Tukey's test; *p<0.05, **p<0.01, and ***p<0.001. F-Dox: free doxorubicin, PLD: pegylated liposomal doxorubicin, PLAD: pegylated liposomal alendronate doxorubicin, NKT: natural killer T-cells.

Besides TAM and MDSC, other myeloid populations such as DC and NK cells play critical roles in modulating immune responses against cancer cells. We found that PLAD also significantly increased the proportion of antigen-presenting dendritic cells (DC1) and decreased the proportion of conventional dendritic cells (DC2) (Figure 5A-B), suggesting a beneficial effect on antitumor immunity since DC2 are often tolerogenic in cancer while DC1 mediate antigen recognition and activation of T cells. PLD and F-Dox did not have any statistically significant effects on DC. In addition, F-Dox decreased NK cells (Figure 5D), suggesting a detrimental effect on antitumor immunity. PLD and PLAD did not induce any significant changes in NK tumor infiltration compared to vehicle control (Figure 5D), however, PLD and PLAD significantly increased the proportion of NK cells that were activated (Figure 5E), suggesting enhancement of antitumor functionality. We next inspected tumor-infiltrating T cells, a heterogeneous population that includes effector T cells such as CD8a^+^ cytotoxic T cells (CTL) and natural killer T cells (NKT), as well as CD4^+^ helper T cells and Tregs. The majority of tumor-infiltrating T cells in this fibrosarcoma model were helper T cells, while CTL comprised fewer than 10% of tumor-infiltrating T cells (Figure 6A-B). PLAD decreased helper T cells as a proportion of total leukocytes, but did not induce significant changes in CTL (Figure 6B). However, the ratio of CD4^+^/CD8a^+^ T cells was lower in both PLD and PLAD treated mice (Figure 6C), which may be indicative of less immunosuppression in the tumor microenvironment. The proportion of FoxP3^+^ regulatory T cells in the CD4^+^ helper population was also decreased in PLAD, PLD, and F-Dox, with the largest effect seen for PLAD (Figure 6E). Although the number of NKT cells were not affected by treatments (Supplemental Figure S3), both PLD and PLAD increased the proportion of NKT cells that were activated (Figure 6F). Taken together, these changes indicate that PLAD, and to a lesser extent PLD, remodeled the tumor microenvironment towards an immune-permissive milieu.

### *In vivo* drug uptake in tumor-dispersed cells

**Figure 7:**
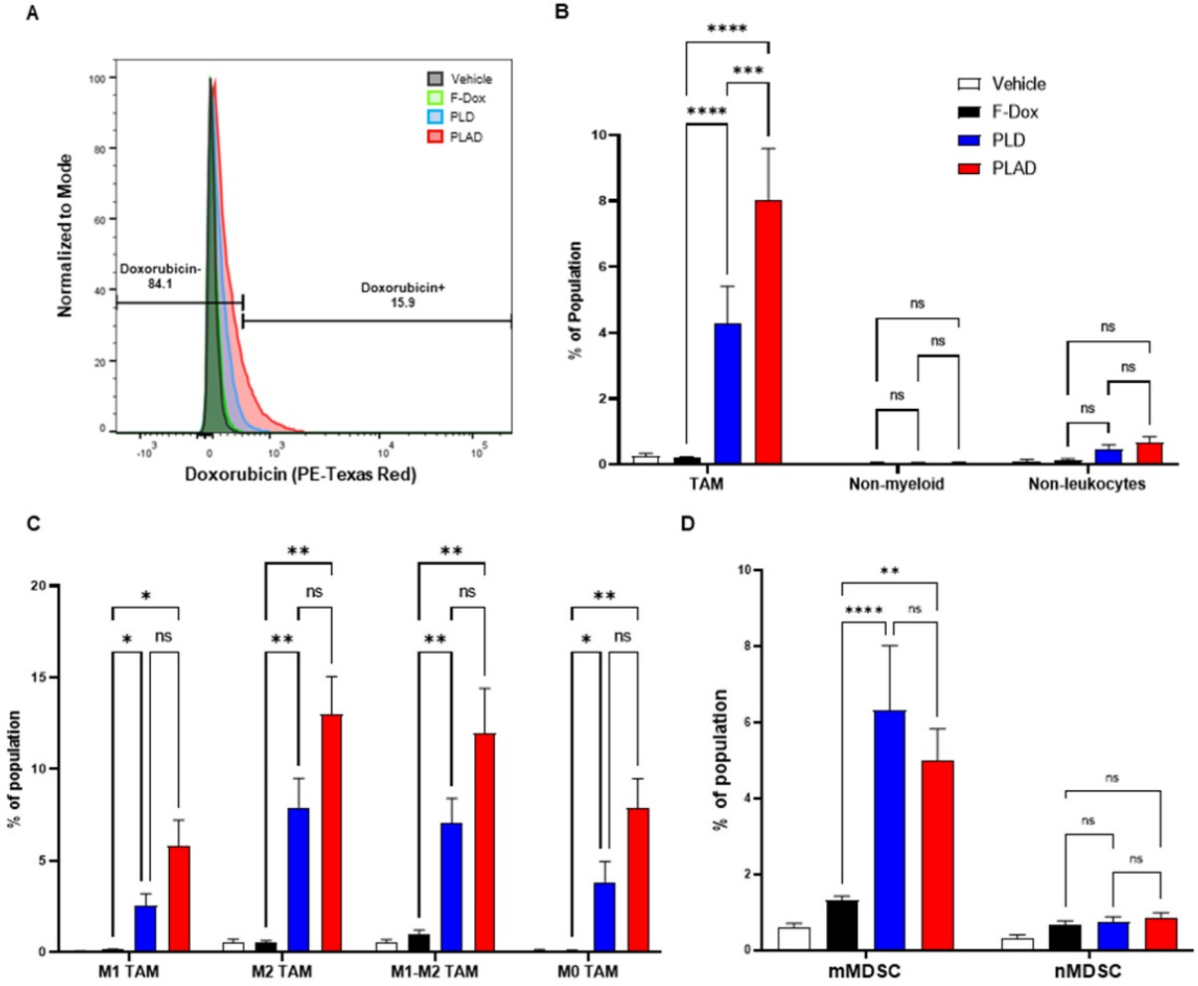
** Liposomal drug delivery significantly increases internalization of doxorubicin by TAM and mMDSC. (A)** Gating strategy for doxorubicin fluorescence. **(B)** Doxorubicin uptake in TAM, non-myeloid leukocytes (i.e., lymphocytes), and non-leukocytes (i.e., tumor and stromal cells). **(C)** Doxorubicin uptake in TAM by polarization state and treatment. **(D)** Doxorubicin uptake in mMDSC and nMDSC. Data are mean with SEM, n=9 for PLAD, PLD and F-Dox, n=5 for vehicle; ANOVA with Tukey's test; *p<0.05, **p<0.01, ***p<0.001, and ****p<0.0001. F-Dox: free doxorubicin, PLD: pegylated liposomal doxorubicin, PLAD: pegylated liposomal alendronate doxorubicin, TAM: tumor associated macrophages, mMDSC: monocytic myeloid derived suppressor cells, nMDSC: neutrophilic myeloid derived suppressor cells.

Our biodistribution study above, and others previously published, showed that PLAD and PLD increased doxorubicin accumulation in tumors compared to F-Dox, but the extent of cellular uptake in different populations of tumor-infiltrating immune cells is unclear. Using multiparameter flow cytometry, we found that there was significant uptake of doxorubicin in TAM from mice treated with PLD and PLAD, with PLAD significantly higher than PLD (Figure 7A-B). There was no significant uptake detected in mice treated with F-Dox, and doxorubicin was not detected in non-myeloid leukocytes (i.e., lymphocytes) or in non-leukocytes (i.e., tumor and stromal cells) for any treatment group (Figure 7A-B). PLAD and PLD delivered doxorubicin to all TAM subpopulations and, although it was not statistically significant, more doxorubicin was delivered by PLAD than by PLD (Figure 7C). Interestingly, M2-polarized TAM and TAM expressing both M1 and M2 markers (M1-M2 TAM) had more uptake than M1-polarized and M0-unpolarized TAM (Figure 7C). PLAD and PLD also delivered doxorubicin to monocytic MDSC, while no significant doxorubicin was detected in neutrophilic MDSC (Figure 7D).

### Fluorescence microscopy of tumor sections

**Figure 8:**
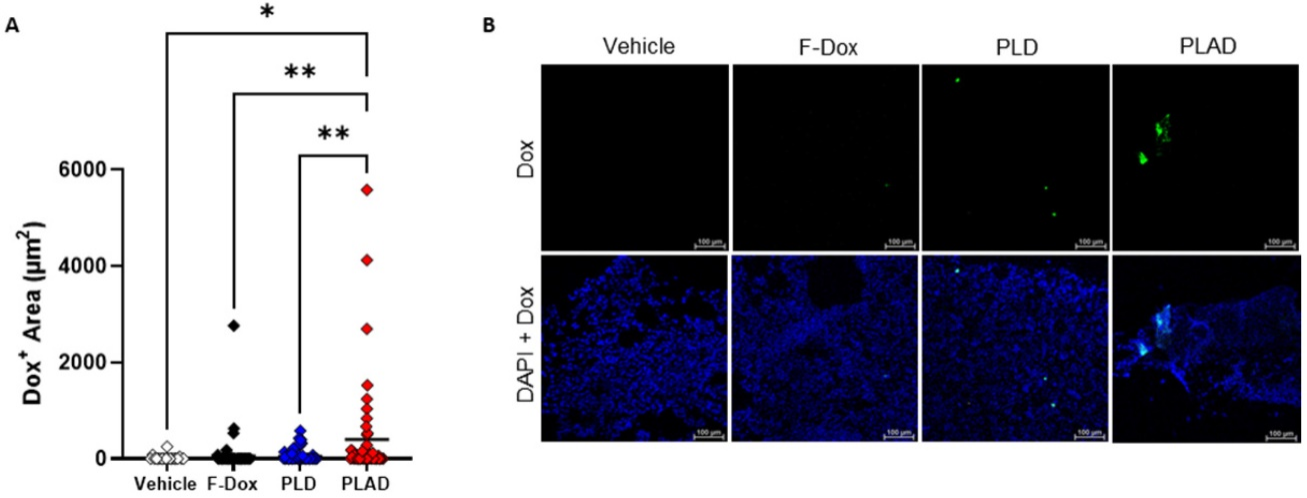
**Doxorubicin accumulation in the tumor by fluorescence microscopy. (A)** Doxorubicin fluorescence in tumor sections showed higher uptake in tumors from PLAD group. Each point is one slide image, 4-11 images/tumor, 23 total animals (PLAD n=7, PLD n=8, F-Dox n=5, and vehicle n=3), bars represent group mean; ANOVA and Dunnett's test; *p<0.05, **p<0.01. **(B)** Representative images shown. F-Dox: free doxorubicin, PLD: pegylated liposomal doxorubicin, PLAD: pegylated liposomal alendronate doxorubicin; DAPI is a nuclear dye.

In tumor sections, significant doxorubicin was detected only in tumors from mice treated with PLAD, while little or no doxorubicin signal was observed in PLD or F-Dox treated mice (Figure 8A-B), corroborating our FACS findings that PLAD delivered more doxorubicin to cells within tumors than PLD and F-Dox. The fact that no significant doxorubicin fluorescence was detected in the PLD group, in contrast to the biodistribution study (Figure 2), suggests that a large fraction of PLD remains extracellular and quenched.

### Antitumor efficacy

**Figure 9:**
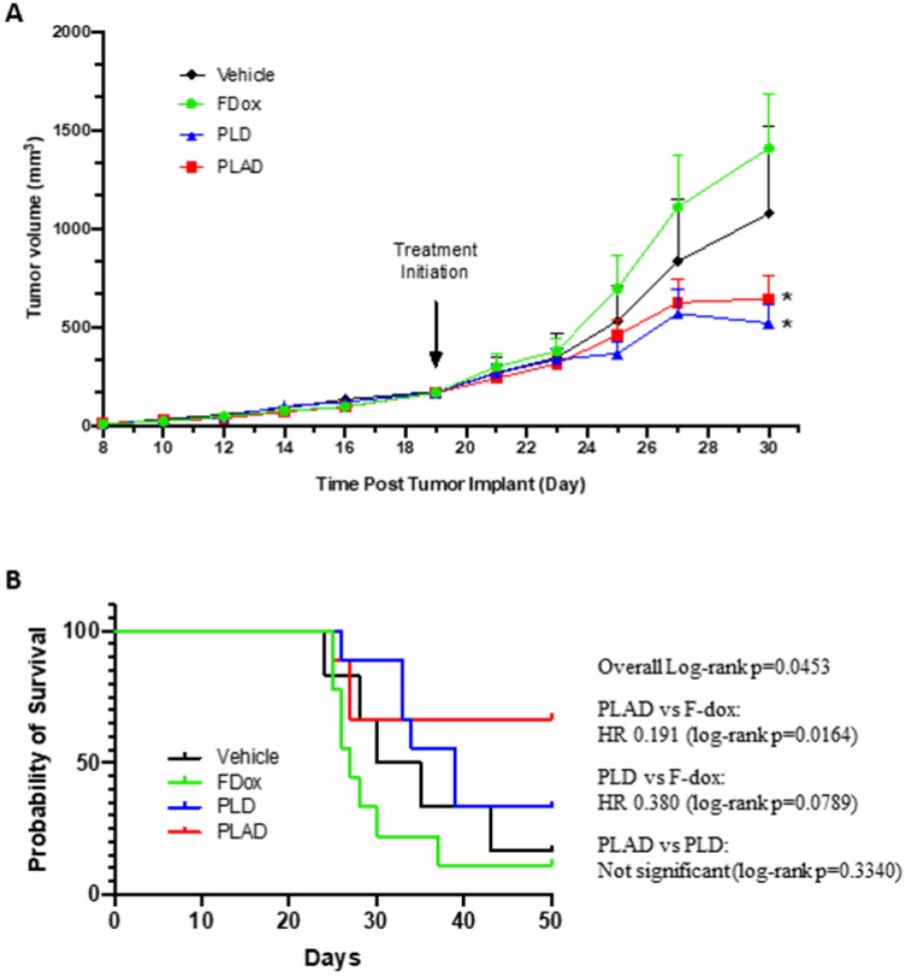
** PLAD and PLD showed superior antitumor efficacy over free doxorubicin in the WEHI-164 fibrosarcoma model. (A)** Tumor growth curves showing group mean with SEM; ANOVA with Dunnett's test; *versus F-Dox, p = 0.0267 and 0.0455, respectively, for PLAD and PLD. **(B)** Kaplan-meier curves for survival endpoint of 5-fold tumor growth; Log-rank tests. PLD, PLAD, and F-Dox, n=9 each group; vehicle n=6. F-Dox: free doxorubicin, PLD: pegylated liposomal doxorubicin, PLAD: pegylated liposomal alendronate doxorubicin.

Tumors were established subcutaneously and allowed to progress to an advanced stage (tumor volume of 150-200 mm^3^) before treatment was initiated. Both PLD and PLAD showed greatly superior antitumor efficacy with significantly increased delay of tumor progression compared to F-Dox (Figure 9A-B). The median time to endpoint (5-fold increase of tumor volume) for F-Dox was 27 days, while for PLD it was 39 days, and it was not reached for PLAD. Kaplan-Meier curves displaying the estimated survival probabilities for this endpoint indicate a significant difference between the survival curves (p=0.0453). For two groups comparison, there was a significant difference between PLAD versus F-Dox (p=0.0164), but the differences between PLD versus F-Dox and between PLAD versus PLD were not statistically significant (p=0.0789 and p=0.3340, respectively). In the PLD and PLAD groups there were 3/9 and 4/9 mice, respectively, with inflamed and necrotic tumors that were withdrawn from the therapeutic study for humane reasons; 33% and 50%, respectively, of these tumors were decreasing in size. Among the F-Dox and vehicle groups, there was only one mouse in the vehicle group with a necrotic tumor that was withdrawn for humane reasons but, unlike PLD and PLAD, this tumor was not shrinking.

## Discussion

It is well-established that the PLD formulation can exploit leaky tumor vasculature to increase doxorubicin delivery to tumors 37-39. Furthermore, radiolabeled PLA has useful imaging properties to select tumors with high EPR effect that are the most likely to respond to nanomedicines 33 and has been shown to increase the number and activation of tumor-infiltrating Vγ9Vδ2 T cells 40, all of which suggest that the co-encapsulated formulation, PLAD, can be a unique nanotheranostic and chemo-immunotherapy platform. We have shown previously in murine breast and lung cancer models 34, and confirmed in the present mouse sarcoma model, that PLAD retains a level of tumor doxorubicin delivery similar to PLD despite increased uptake in spleen. In this study, we confirm the superior drug delivery of the liposomal formulations over free drug and demonstrate important differences in the cellular distribution of PLAD and PLD within tumors with overall greater cell uptake of PLAD. It is likely that a large fraction of PLD remains extracellular or intra-vascular with quenched fluorescence, thus accounting for the discrepancy between the negligible fluorescence in tumor sections of PLD treated mice and the quantitation of doxorubicin in tumors in the biodistribution study. In addition, our study also shows a significant shift in the tumor-infiltrating cell populations in liposome-treated mice, which was much more striking with PLAD than with PLD. These data support the contention that the presence of alendronate in PLAD confers unique immunologic and pharmacologic properties that may provide a therapeutic advantage over PLD particularly in this sarcoma model. Despite this, our therapeutic study did not reveal a major difference in outcome between PLAD and PLD, possibly due to early withdrawal in the PLAD group for reasons unrelated to excessive tumor burden. Interestingly, PLAD treatment was associated with higher incidence of inflamed and necrotic tumors that were shrinking compared to the other treatment groups, suggesting a possible link between treatment-induced immune responses and antitumor efficacy. Further therapeutic studies with a larger sample size may help to clarify this issue.

Historically, the majority of biodistribution studies for nanoparticle-delivered drugs (including liposomal drugs) focus on quantitating drug concentrations at the level of organs/tissues 41-43, and very few probe cell-type specific drug uptake beyond tumor cells and macrophages 44. Kupffer cells, splenic macrophages, and, to a minor extent, circulating monocytes (precursors of macrophages) have been shown to be key players in the clearance of systemically administered liposomes and other nanoparticle delivery systems 45-47, but the specific role of TAM in the pharmacokinetics of nanoparticle-delivered drugs has not been fully elucidated 28, 48, 49. Miller and colleagues reported that a PLGA-PEG based polymeric nanoparticle platform directed cellular uptake toward TAM and that TAM served as a reservoir for drug release to local tumor cells 50. Importantly, we found that PLD and specially PLAD not only directed cellular drug uptake to TAM, but also were associated with M1 and M2 polarization. We further noticed that there was significant uptake of liposomal drugs in monocytic MDSC. MDSC are a heterogenous population of myeloid progenitor cells that are expanded and found in large numbers in tissue under pathological conditions, such as cancer and chronic inflammatory diseases. MDSC are characterized as neutrophilic or monocytic, and the latter subtype has protumoral activity including suppression of antitumor T cell responses and direct stimulation of tumor growth and metastasis, and can give rise to TAM 51, 52. Our findings reveal not only substantial differences in TAM drug uptake between PLD and PLAD, but also suggest a link between liposomal drug uptake and immunosuppressive functionality of TAM and MDSC. Liposome uptake and the specific cargo (PLD or PLAD) cause functional polarization of TAM and further changes in the tumor microenvironment with implications on the therapeutic performance of liposomal drugs versus F-Dox that go beyond the simple increase in drug delivery conferred by the EPR effect.

Drug-free nanoparticles have immune modulatory activity 29, 53, 54, although this effect is largely viewed as secondary when a drug cargo is present. However, while the direct antitumor activity of nanoparticle-delivered drugs has been well emphasized by many studies, their impact on the tumor immunologic milieu is not fully appreciated 49. Herein, we systematically evaluated the impact of PLD and PLAD on the tumor immunologic milieu. Besides doxorubicin, PLAD contains alendronate, a potent amino-bisphosphonate, which upon encapsulation in liposomes, has macrophage suppressive effects. In addition, alendronate induces expression of non-peptidic phospho-antigens that activate a specific subpopulation of human T cells expressing the Vγ9Vδ2 TCR with direct tumoricidal effects 55. We observed that the free formulation of doxorubicin induced moderate changes in the tumor microenvironment. Some of these effects, such as a decrease in tumor infiltration of Tregs, could promote antitumor immune responses, while others, such as a decrease in M1 TAM and a decrease in NK cells, could be detrimental to antitumor immune responses. When doxorubicin is encapsulated in liposomes (i.e., PLD), we observed that it induced significant changes in number and functionality of tumor-infiltrating TAM, MDSC, Treg, NKT, and NK cells that are consistent with enhanced antitumor immune responses in the tumor microenvironment. The co-encapsulation of alendronate with doxorubicin (i.e., PLAD) generally enhanced these effects over PLD, but was not statistically significant. These immune modulatory effects are reflected in the therapeutic study which showed that free doxorubicin did not inhibit tumor growth, while PLAD and PLD showed significant tumor growth inhibition. The total lack of efficacy that we observed for free doxorubicin was rather unexpected. One possible explanation for this is that in our study, tumors were well advanced when treatment was started, and these larger tumors have developed profound immunosuppression in the tumor microenvironment making them both less responsive to free doxorubicin and more responsive to the immune modulatory effects of PLD and PLAD. In contrast, if treatment is started when tumors are very small, the tumor immunologic milieu is not yet fully developed, and free cytotoxic therapy alone may be efficacious.

The immune modulatory activity of PLAD, and to a lesser extent PLD, favor an immune-permissive microenvironment suggesting that combination with immune checkpoint inhibitors, such as blockading antibodies against programmed cell death protein 1 (PD-1), could result in synergistic anticancer efficacy. There are a few examples of drug co-encapsulation in nanomedicine formulations that include anthracycline drugs 35, 56 and one of these formulations (Vyxeos^TM^) has reached clinical approval for the treatment of acute myelogenous leukemia 57. One unique feature of PLAD is that the co-encapsulated agents belong to totally different chemical categories and have totally different mechanisms of actions (cytotoxicity and immunomodulation), thus reducing the risk of an overlap of toxic effects (e.g., myelosuppression, cardiotoxicity), an important factor that may facilitate its clinical translatability. Another specific property of PLAD, absent in PLD, is the strong metal-chelating properties of alendronate-containing liposomes which enable follow-up of the liposome biodistribution by nuclear imaging 33, 40.

## Conclusion

We showed that liposomal doxorubicin delivery not only alters pharmacokinetics, but also dramatically changes the immune modulatory activity of the drug cargo. Together, our data support liposomes as platforms for combinatorial drug therapy and immunotherapy to selectively modulate TAM and mMDSC within the tumor microenvironment. Furthermore, our study reveals that alendronate has the potential to boost these effects when co-encapsulated with doxorubicin in the PLAD formulation. When considering the added value of the PET imaging properties of alendronate complexes with positron emitters, PLAD is an attractive nanotheranostic tool with a strong rationale for combined therapeutic use with immune check-point inhibitors and/or adoptive T cell therapy.

## Supplementary Material

Supplementary figures and tables.

## References

[1] Edwards TM, Duran MS, Meeker TM (2017). Congenital Infantile Fibrosarcoma in the Premature Infant. Adv Neonatal Care.

[2] Folpe AL (2014). Fibrosarcoma: a review and update. Histopathology.

[3] Das B, Jain N, Mallick B (2021). piR-39980 mediates doxorubicin resistance in fibrosarcoma by regulating drug accumulation and DNA repair. Commun Biol.

[4] Hoefkens F, Dehandschutter C, Somville J, Meijnders P, Van Gestel D (2016). Soft tissue sarcoma of the extremities: pending questions on surgery and radiotherapy. Radiat Oncol.

[5] Augsburger D, Nelson PJ, Kalinski T, Udelnow A, Knosel T, Hofstetter M (2017). Current diagnostics and treatment of fibrosarcoma -perspectives for future therapeutic targets and strategies. Oncotarget.

[6] Johnson-Arbor K, Dubey R (2022). Doxorubicin. StatPearls. Treasure Island (FL).

[7] Ratan R, Patel SR (2016). Chemotherapy for soft tissue sarcoma. Cancer.

[8] Savani M, Murugan P, Skubitz KM (2019). Long-term cure of soft tissue sarcoma with pegylated-liposomal doxorubicin after doxorubicin and ifosfamide failure. Clin Sarcoma Res.

[9] Bramwell VH, Anderson D, Charette ML, Sarcoma Disease Site G Doxorubicin-based chemotherapy for the palliative treatment of adult patients with locally advanced or metastatic soft tissue sarcoma. Cochrane Database Syst Rev. 2003: CD003293.

[10] Tian Z, Yang Y, Yang Y, Zhang F, Li P, Wang J (2020). High cumulative doxorubicin dose for advanced soft tissue sarcoma. BMC Cancer.

[11] Burade V, Bhowmick S, Maiti K, Zalawadia R, Jain D, Rajamannar T (2017). Comparative plasma and tissue distribution of Sun Pharma's generic doxorubicin HCl liposome injection versus Caelyx((R)) (doxorubicin HCl liposome injection) in syngeneic fibrosarcoma-bearing BALB/c mice and Sprague-Dawley rats. Cancer Chemother Pharmacol.

[12] Shi Y, van der Meel R, Chen X, Lammers T (2020). The EPR effect and beyond: Strategies to improve tumor targeting and cancer nanomedicine treatment efficacy. Theranostics.

[13] La-Beck NM, Gabizon AA (2017). Nanoparticle Interactions with the Immune System: Clinical Implications for Liposome-Based Cancer Chemotherapy. Front Immunol.

[14] Saggioro M, D'Angelo E, Bisogno G, Agostini M, Pozzobon M (2020). Carcinoma and Sarcoma Microenvironment at a Glance: Where We Are. Front Oncol.

[15] Koo J, Hayashi M, Verneris MR, Lee-Sherick AB (2020). Targeting Tumor-Associated Macrophages in the Pediatric Sarcoma Tumor Microenvironment. Front Oncol.

[16] Roulleaux Dugage M, Nassif EF, Italiano A, Bahleda R (2021). Improving Immunotherapy Efficacy in Soft-Tissue Sarcomas: A Biomarker Driven and Histotype Tailored Review. Front Immunol.

[17] Germano G, Frapolli R, Belgiovine C, Anselmo A, Pesce S, Liguori M (2013). Role of macrophage targeting in the antitumor activity of trabectedin. Cancer Cell.

[18] Chen L, Oke T, Siegel N, Cojocaru G, Tam AJ, Blosser RL (2020). The Immunosuppressive Niche of Soft-Tissue Sarcomas is Sustained by Tumor-Associated Macrophages and Characterized by Intratumoral Tertiary Lymphoid Structures. Clin Cancer Res.

[19] Fujiwara T, Healey J, Ogura K, Yoshida A, Kondo H, Hata T (2021). Role of Tumor-Associated Macrophages in Sarcomas. Cancers (Basel).

[20] Larionova I, Cherdyntseva N, Liu T, Patysheva M, Rakina M, Kzhyshkowska J (2019). Interaction of tumor-associated macrophages and cancer chemotherapy. Oncoimmunology.

[21] Salmaninejad A, Valilou SF, Soltani A, Ahmadi S, Abarghan YJ, Rosengren RJ (2019). Tumor-associated macrophages: role in cancer development and therapeutic implications. Cell Oncol (Dordr).

[22] Kloepper J, Riedemann L, Amoozgar Z, Seano G, Susek K, Yu V (2016). Ang-2/VEGF bispecific antibody reprograms macrophages and resident microglia to anti-tumor phenotype and prolongs glioblastoma survival. Proc Natl Acad Sci U S A.

[23] Mantovani A, Marchesi F, Malesci A, Laghi L, Allavena P (2017). Tumour-associated macrophages as treatment targets in oncology. Nat Rev Clin Oncol.

[24] Saerens M, Brusselaers N, Rottey S, Decruyenaere A, Creytens D, Lapeire L (2021). Immune checkpoint inhibitors in treatment of soft-tissue sarcoma: A systematic review and meta-analysis. Eur J Cancer.

[25] Grimaldi A, Cammarata I, Martire C, Focaccetti C, Piconese S, Buccilli M (2020). Combination of chemotherapy and PD-1 blockade induces T cell responses to tumor non-mutated neoantigens. Commun Biol.

[26] Nakamura T, Sudo A (2022). The Role of Trabectedin in Soft Tissue Sarcoma. Front Pharmacol.

[27] Wanderley CW, Colon DF, Luiz JPM, Oliveira FF, Viacava PR, Leite CA (2018). Paclitaxel Reduces Tumor Growth by Reprogramming Tumor-Associated Macrophages to an M1 Profile in a TLR4-Dependent Manner. Cancer Res.

[28] Zeisberger SM, Odermatt B, Marty C, Zehnder-Fjallman AH, Ballmer-Hofer K, Schwendener RA (2006). Clodronate-liposome-mediated depletion of tumour-associated macrophages: a new and highly effective antiangiogenic therapy approach. Br J Cancer.

[29] Rajan R, Sabnani MK, Mavinkurve V, Shmeeda H, Mansouri H, Bonkoungou S (2018). Liposome-induced immunosuppression and tumor growth is mediated by macrophages and mitigated by liposome-encapsulated alendronate. Journal of controlled release: official journal of the Controlled Release Society.

[30] Thompson K, Roelofs AJ, Jauhiainen M, Monkkonen H, Monkkonen J, Rogers MJ (2010). Activation of gammadelta T cells by bisphosphonates. Adv Exp Med Biol.

[31] Sharpe M, Noble S, Spencer CM (2001). Alendronate: an update of its use in osteoporosis. Drugs.

[32] La-Beck NM, Liu X, Shmeeda H, Shudde C, Gabizon AA (2021). Repurposing amino-bisphosphonates by liposome formulation for a new role in cancer treatment. Semin Cancer Biol.

[33] Edmonds S, Volpe A, Shmeeda H, Parente-Pereira AC, Radia R, Baguna-Torres J (2016). Exploiting the Metal-Chelating Properties of the Drug Cargo for *In vivo* Positron Emission Tomography Imaging of Liposomal Nanomedicines. ACS Nano.

[34] Shmeeda H, Amitay Y, Gorin J, Tzemach D, Mak L, Stern ST (2016). Coencapsulation of alendronate and doxorubicin in pegylated liposomes: a novel formulation for chemoimmunotherapy of cancer. J Drug Target.

[35] Gabizon A, Ohana P, Amitay Y, Gorin J, Tzemach D, Mak L (2021). Liposome co-encapsulation of anti-cancer agents for pharmacological optimization of nanomedicine-based combination chemotherapy. Cancer Drug Resist.

[36] Goren D, Horowitz AT, Zalipsky S, Woodle MC, Yarden Y, Gabizon A (1996). Targeting of stealth liposomes to erbB-2 (Her/2) receptor: *in vitro* and *in vivo* studies. Br J Cancer.

[37] Gabizon A, Papahadjopoulos D (1988). Liposome formulations with prolonged circulation time in blood and enhanced uptake by tumors. Proc Natl Acad Sci U S A.

[38] Harrington KJ, Mohammadtaghi S, Uster PS, Glass D, Peters AM, Vile RG (2001). Effective targeting of solid tumors in patients with locally advanced cancers by radiolabeled pegylated liposomes. Clin Cancer Res.

[39] Symon Z, Peyser A, Tzemach D, Lyass O, Sucher E, Shezen E (1999). Selective delivery of doxorubicin to patients with breast carcinoma metastases by stealth liposomes. Cancer.

[40] Man F, Lim L, Volpe A, Gabizon A, Shmeeda H, Draper B (2019). *In vivo* PET Tracking of (89)Zr-Labeled Vgamma9Vdelta2 T Cells to Mouse Xenograft Breast Tumors Activated with Liposomal Alendronate. Mol Ther.

[41] Gabizon A, Price DC, Huberty J, Bresalier RS, Papahadjopoulos D (1990). Effect of liposome composition and other factors on the targeting of liposomes to experimental tumors: biodistribution and imaging studies. Cancer Res.

[42] Harrington KJ, Rowlinson-Busza G, Syrigos KN, Abra RM, Uster PS, Peters AM (2000). Influence of tumour size on uptake of(111)ln-DTPA-labelled pegylated liposomes in a human tumour xenograft model. Br J Cancer.

[43] Huang SK, Lee KD, Hong K, Friend DS, Papahadjopoulos D (1992). Microscopic localization of sterically stabilized liposomes in colon carcinoma-bearing mice. Cancer Res.

[44] Corbo C, Molinaro R, Taraballi F, Toledano Furman NE, Sherman MB, Parodi A (2016). Effects of the protein corona on liposome-liposome and liposome-cell interactions. Int J Nanomedicine.

[45] Daemen T, Hofstede G, Ten Kate MT, Bakker-Woudenberg IA, Scherphof GL (1995). Liposomal doxorubicin-induced toxicity: depletion and impairment of phagocytic activity of liver macrophages. Int J Cancer.

[46] Caron WP, Lay JC, Fong AM, La-Beck NM, Kumar P, Newman SE (2013). Translational studies of phenotypic probes for the mononuclear phagocyte system and liposomal pharmacology. J Pharmacol Exp Ther.

[47] La-Beck NM, Zamboni BA, Gabizon A, Schmeeda H, Amantea M, Gehrig PA (2012). Factors affecting the pharmacokinetics of pegylated liposomal doxorubicin in patients. Cancer Chemother Pharmacol.

[48] Moghimi SM, Szebeni J (2003). Stealth liposomes and long circulating nanoparticles: critical issues in pharmacokinetics, opsonization and protein-binding properties. Prog Lipid Res.

[49] La-Beck NM, Liu X, Wood LM (2019). Harnessing Liposome Interactions With the Immune System for the Next Breakthrough in Cancer Drug Delivery. Front Pharmacol.

[50] Miller MA, Zheng YR, Gadde S, Pfirschke C, Zope H, Engblom C (2015). Tumour-associated macrophages act as a slow-release reservoir of nano-therapeutic Pt(IV) pro-drug. Nat Commun.

[51] Gabrilovich DI, Nagaraj S (2009). Myeloid-derived suppressor cells as regulators of the immune system. Nat Rev Immunol.

[52] Gabrilovich DI (2017). Myeloid-Derived Suppressor Cells. Cancer Immunol Res.

[53] Ilinskaya AN, Dobrovolskaia MA (2014). Immunosuppressive and anti-inflammatory properties of engineered nanomaterials. Br J Pharmacol.

[54] Alving CR (1992). Immunologic aspects of liposomes: presentation and processing of liposomal protein and phospholipid antigens. Biochim Biophys Acta.

[55] Clezardin P (2011). Bisphosphonates' antitumor activity: an unravelled side of a multifaceted drug class. Bone.

[56] Shuhendler AJ, Prasad P, Zhang RX, Amini MA, Sun M, Liu PP (2014). Synergistic nanoparticulate drug combination overcomes multidrug resistance, increases efficacy, and reduces cardiotoxicity in a nonimmunocompromised breast tumor model. Molecular pharmaceutics.

[57] Lancet JE, Uy GL, Cortes JE, Newell LF, Lin TL, Ritchie EK (2018). CPX-351 (cytarabine and daunorubicin) Liposome for Injection Versus Conventional Cytarabine Plus Daunorubicin in Older Patients With Newly Diagnosed Secondary Acute Myeloid Leukemia. Journal of clinical oncology: official journal of the American Society of Clinical Oncology.

