# Supplementary material for: Comparative effects of free doxorubicin, liposome encapsulated doxorubicin and liposome co-encapsulated alendronate and doxorubicin (PLAD) on the tumor immunologic milieu in a mouse fibrosarcoma model
Source: Nanotheranostics. 2022 Sep 1;6(4):451–64. doi: 10.7150/ntno.75045 (PMC9461478; doi:10.7150/ntno.75045)
Supplement: Supplementary file 1 — Supplementary figures and tables. [file ntnov06p0451s1.pdf]

PLAD Batch	Size (nm)	PDI	Zeta pot. (mV)	Osm. mOsm /kg	pH	ALD (mg/g)	Cholesterol (mg/g)	DSPE-PEG_{2000} (mg/g)	HSPC (mg/g)	DOX-HCl (mg/g)
Levco 3	110.3	0.058	-12.13	317	7.1	0.5	1.62	1.36	4.6	0.9
Levco 2	99.8	0.028	-13.41	291	6.7	0.6	1.62	1.21	4.3	0.9

Supplemental Table S1: Characteristics of PLAD batches used in this study. All values fall within the pre-specified acceptable range. The potency of the batch is labeled as 1 mg/mL (acceptable range 0.9-1.1) in DOX HCl equivalents, although the actual result is 0.9 mg/g or mL.

**Myeloid Panel (TAM, MDSC, DC)**		
**Antigen**	**Antibody Clone**	**Fluorophore**
CD45	30-F11	AF700
CD11b	M1/70	AF647
Gr1	RB6-8C5	AF488
F4/80	BM8	BV421
CD11c	N418	APC-Cy7
CD8a	53-6.7	PerCP-Cy5.5
CD206	C068C2	BV650
MHC-II	M5/114.15.2	BV510
(Viability dye)	(Zombie Yellow)	(BV570)
(Doxorubicin)	-	(PE-TR)

Supplemental Table S2: Reagents for myeloid panel

**CTL, NK, NKT Panel**		
**Antigen**	**Antibody Clone**	**Fluorophore**
CD45	30-F11	AF700
CD3	17A2 (T and NKT)	APC-Cy7
CD4	GK1.5	AF488
CD8a	53-6.7	PerCP-Cy5.5
CD49b	DX5	AF647
NKp46	29A1.4	BV510
CD69	H1.2F3	PE-CF594
CTLA-4	UC10-4B9	PE
PD-1	29F.1A12	BV421
Viability dye	(Zombie Yellow)	(BV570)

Supplemental Table S3: Reagents for CTL, NK and NKT panel

**Treg Panel**		
**Antigen**	**Antibody Clone**	**Fluorophore**
CD45	30-F11	AF700
CD4	GK1.5	AF488
CD25	PC61	AF647
FoxP3 (nuclear)	MF-14	BV421
Viability dye	(Zombie Yellow)	(BV570)

Supplemental Table S4: Reagents for Treg panel

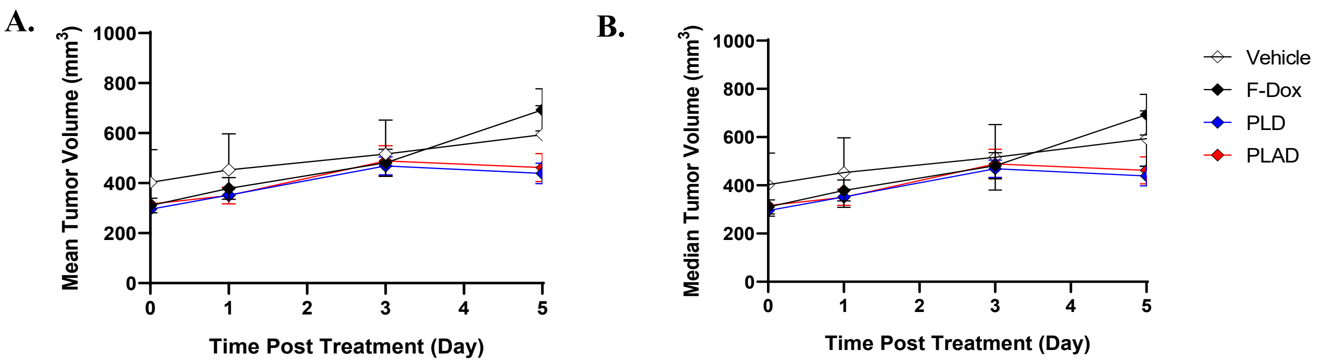

Supplemental Figure S1: The (A) mean and (B) median tumor volumes of different treatment groups (post treatment) were not statistically different in significance at any timepoint until tissue collection. Data analysis: Two-Way ANOVA (Dunnett's multiple comparisons); GraphPad Prism 9.3.1 (471).

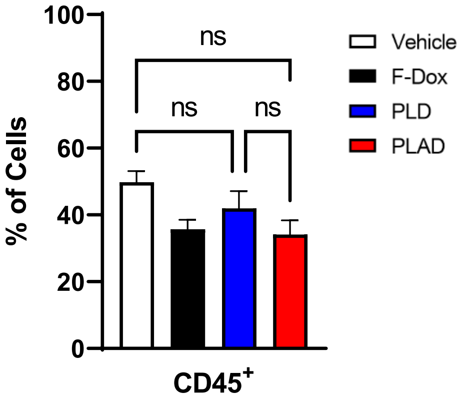

Supplemental Figure S2: There was no statistically significant difference in overall numbers of tumor-infiltrating leukocytes between the treatment groups. Data analysis: One-Way ANOVA (Tukey's multiple comparisons); GraphPad Prism 9.3.1 (471).

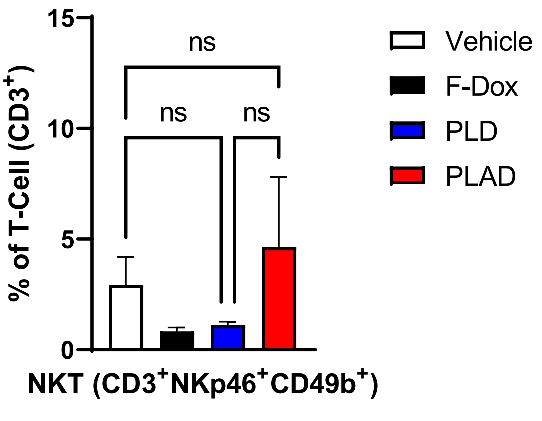

Supplemental Figure S3: The proportions of NKT cells out of total T-cells are not significantly different between treatment groups. Data analysis: One-way ANOVA (Tukey's multiple comparison); GraphPad Prism 9.3.1 (471).

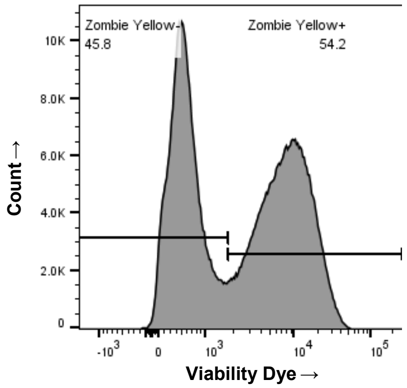

Supplemental Figure S4: Gating strategy for viable cells.
